# Osteochondroma of Distal Femur Managed With Complete Excision: A Case Report

**DOI:** 10.7759/cureus.51714

**Published:** 2024-01-05

**Authors:** Ousama Jelti, Oussama El Alaoui, Adnane Lachkar, Najib Abdeljaouad, Hicham Yacoubi

**Affiliations:** 1 Orthopaedics, Mohammed VI University Hospital, Oujda, MAR; 2 Orthopaedics and Traumatology, Centre Hospitalier Universitaire (CHU) Mohammed VI, Oujda, MAR; 3 Traumatology and Orthopedics, Faculty of Medicine and Pharmacy, Mohammed VI University Hospital, Mohammed First University, Oujda, MAR

**Keywords:** mri, surgery, distal femur, exostosis, osteochondroma

## Abstract

Osteochondromas are benign bone tumors that usually occur between the ages of 10 and 30, with no marked gender preference. These lesions result from the separation of the epiphyseal growth plate and are categorized as growth plate development abnormalities rather than true neoplasms. It is important to note that long-term solitary osteochondromas can evolve into osteosarcomas, with chondrosarcoma being the most common among them. However, the risk of recurrence is considerably reduced if the tumor is completely resected from its original site, with no residual perichondrium or cartilage cap left in place. In this context, a 29-year-old man with osteochondroma in the distal femur was successfully treated with complete resection, showing a favorable evolution.

## Introduction

Osteochondromas are the most common benign bone tumours, accounting for 20-50% of benign bone tumours and 9% of all bone tumours [1,2]. They are developmental deformities rather than true tumours, originating in the outer layer of the bone [3]. Genetic transmission is autosomal dominant and can result in either multiple growths or isolated lesions [4]. These abnormalities usually appear during the phase of rapid skeletal growth and cease to progress once maturity is reached [4]. Patients frequently report increased volume and aesthetic alterations [5]. The lesion usually presents a mushroom-shaped configuration, and it affects the enlarged part of long bones such as the femur and tibia [6]. It mainly affects bones that form by enchondral ossification and rarely those that develop by intramembranous ossification, such as the scapula, pubis, clavicle, and ribs [7]. Although the diagnosis is usually established by plain radiographs, computed tomography (CT) and magnetic resonance imaging (MRI) can provide alternative diagnostic modalities. Surgical excision remains a reliable and effective method of treatment, with consistent results and pain relief.

## Case presentation

We present the case of a 29-year-old young man who consulted us for pain and bone swelling above the left knee joint, which had persisted for 10 years. At the outset, the swelling presented as compact, painless, and featured a firm osseous texture. No associated pain or limitations in knee movement were observed. Nevertheless, over the past two years, pain has surfaced, accompanied by a gradual increase in the swelling. Additionally, the patient reported pain on knee flexion beyond 100 degrees. No treatment had been undertaken since the swelling was discovered, and the patient reported no alteration in general condition or history of similar bony swellings elsewhere in the body.

**Figure 1:**
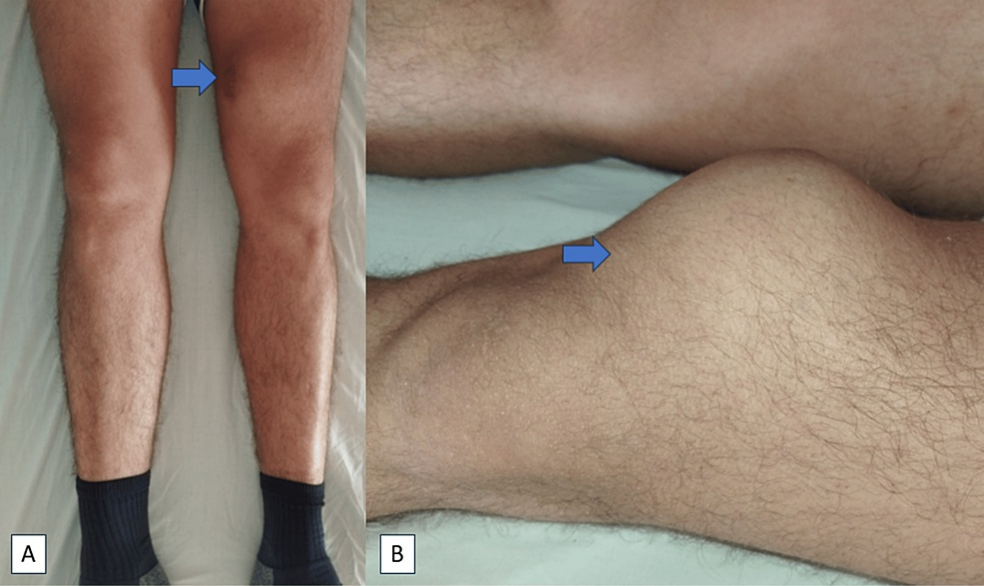
Clinical picture of tumour.

Clinical examination revealed an oval-shaped osseous mass originating from the anteromedial aspect of the lower end of the left femur. The skin covering the mass was tight but intact. Upon palpation, the mass exhibited tenderness, depth, an irregular surface, hard consistency, and immobility. Palpation along the lateral line of the knee joint suggested a bony swelling originating from the metaphyseal-diaphyseal zone of the femur. The dimensions of the mass measured approximately 6 cm by 5 cm. No indications of neurovascular compression were noted. The left knee's range of motion was painless within the span of 0-100 degrees. Nevertheless, surpassing 100 degrees in knee flexion induced pain and a stretching sensation in the skin overlying the osseous mass. Clinical assessments of the ligaments and menisci surrounding the knee were within normal parameters (Figure 1).

**Figure 2:**
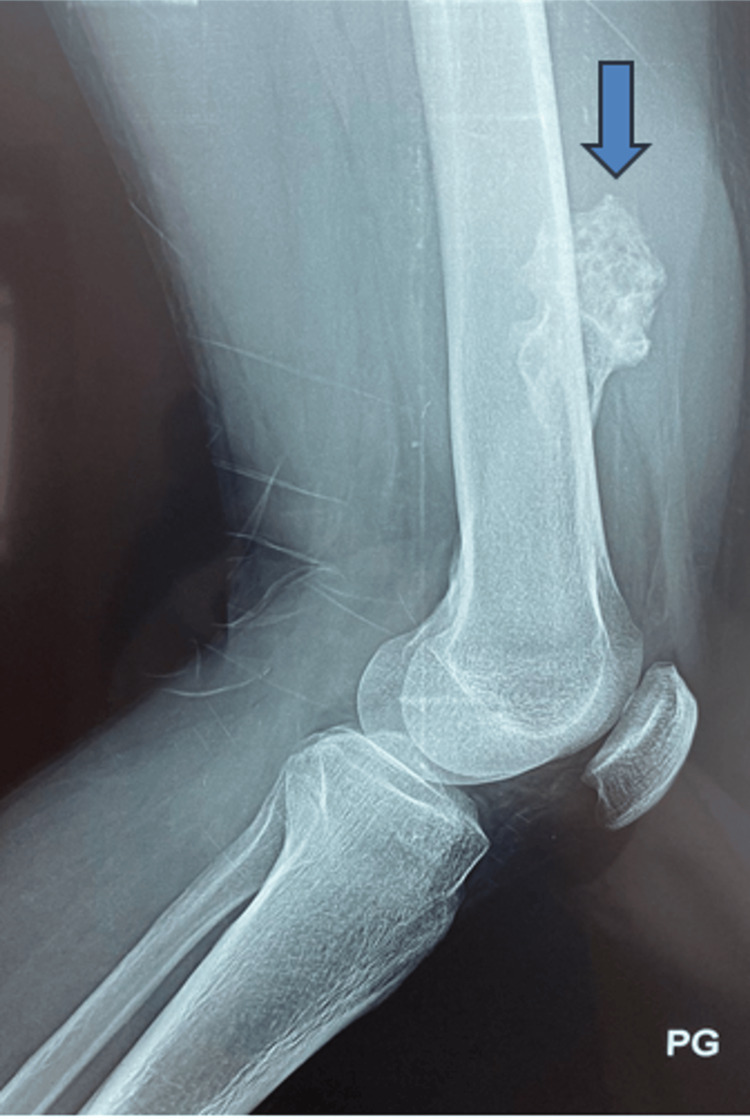
Standard radiograph before osteochondroma resection.

Conventional radiographic images of the left femur, encompassing the knee joint, illustrated a pedunculated osseous mass originating from the anteromedial region of the lower extremity of the left femur. The tumor demonstrated continuous alignment with the femur (Figure 2).

**Figure 3:**
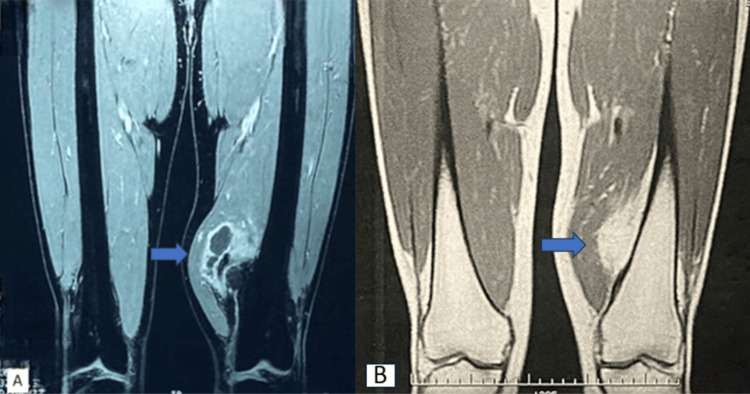
MRI of the left thigh showing a well-limited mass in the anterior compartment of the thigh, in continuity with the medial cortex of the femur, displacing the vastus medialis; hypointense in T1 (A) and hyperintense in T2 (B) with peripheral reassessment.

MRI revealed a well-limited mass, measuring 56x65x75mm, on the lower extremity of the left femur in the anterior thigh compartment, in continuity with the medial cortex of the femur, displacing the vastus medialis, T1 hyposignal, and T2 hyperhypersignal with peripheral and septal enhancement after injection of contrast medium, and this mass respects the femoral pedicle (Figure 3).

**Figure 4:**
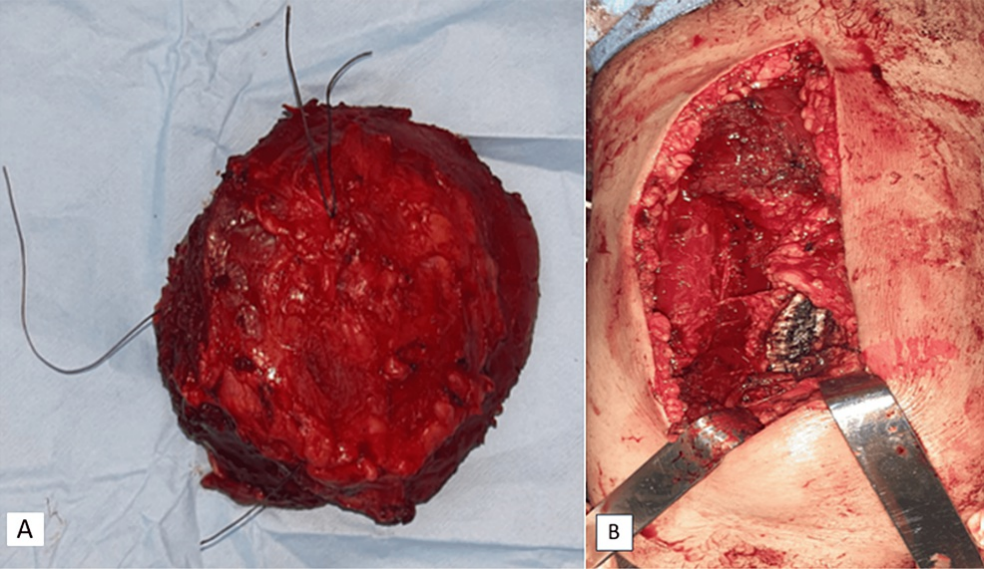
Total excision of osteochondroma.

The patient underwent surgery using an anteromedial approach to the distal femur, during which the tumor was exposed and resected en bloc (Figure 4).

**Figure 5:**
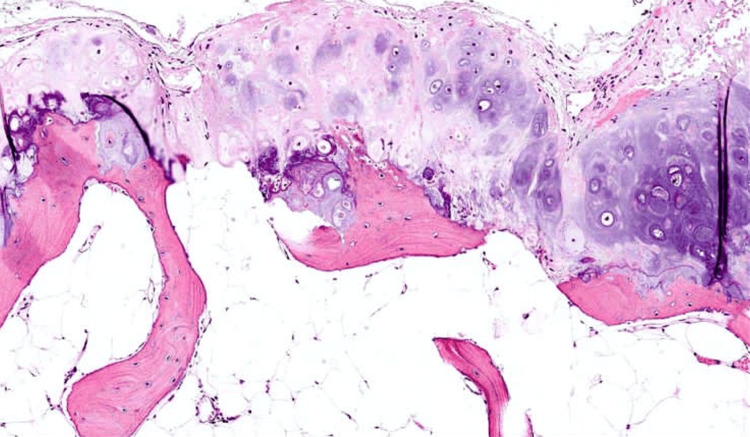
The microphotograph of the lesion reveals an osteocartilaginous proliferation consisting of an outer cartilaginous cap, organized into cell-sparse lobules and housing small cells with hyperchromatic, non-atypical nuclei. The cartilaginous cap is continued by regular bone trabeculae.

The histopathological examination revealed a cartilaginous proliferation with mild to moderate cellularity, characterized by regular chondrocytes exhibiting no cytonuclear atypia, hyperchromatic nuclei, and generally retracted cytoplasm. The cartilaginous proliferation is peripherally delineated by the presence of a fibrous capsule. Additionally, calcifications are observed in association with this cartilaginous proliferation (Figure 5).

**Figure 6:**
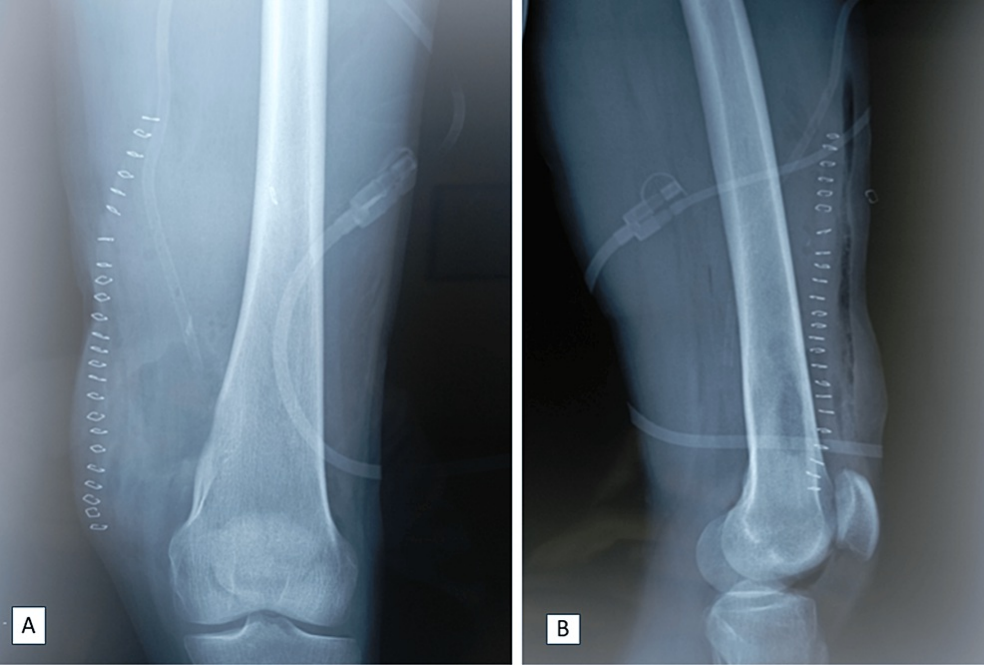
Postoperative X-ray.

Postoperative standard radiographs confirmed complete resection of the tumor (Figure 6).

The patient quickly regained his independence, walking unassisted immediately after surgery, and was discharged from the hospital. After a one-year follow-up, no recurrence was observed.

## Discussion

Osteocartilaginous exostoses are bony protuberances enveloped by a cartilaginous layer, appearing only during the growth period. Although their congenital origin brings them closer to hamartomas, they are categorized as benign bone tumors. These outgrowths, also known as osteochondromas, account for 40% of benign tumors and 10% of all primary bone tumors [8]. Two clinical forms have been identified: solitary exostoses and exostosing disease [9]. The variant with multiple exostoses is identified as an autosomal dominant condition referred to as hereditary multiple exostoses (HME). This condition is characterized by the development of numerous bony protrusions on the epiphyses [10].

Osteochondromas are frequently observed in adolescents and more rarely in newborns. In the case of solitary exostosis, there is no disparity between the sexes. On the other hand, an exostosing disease more frequently affects men. These tumors show a marked preference for the metaphyseal side of the growth plate, which is in full activity. Solitary exostoses are often asymptomatic and are frequently discovered incidentally on radiological examination, rarely causing vascular or neurological complications. Vascular complications, although exceptional, can occur with solitary osteochondromas. Complications associated with HME encompass deformities, fractures, vascular changes, pocket formation, malignant transformation, and neurological sequelae. Osteochondromas can apply direct pressure on arteries, leading to circulatory obstruction and promoting thrombosis. Exostoses affecting the deep venous system contribute to 5% of vascular complications. These diverse vascular lesions are clinically suspected and confirmed through CT angiography, which remains the gold standard [11-13]. Neurological complications vary depending on the location of the exostoses. They manifest as neuropathy, radiculopathy, or spinal cord compression, attributed to exostoses situated around the knee and in proximity to the spinal cord [14].

Frequently, X-rays and CT scans deliver precise diagnostic insights, facilitating the anatomical characterization of the lesion [15]. In imaging, osteochondromas typically present as pedicles or projections resembling sessile bone. Cancellous and cortical structures exhibit a close association with normal bone. The cartilage shadow is distinctly recognizable at the apex of the tumor, featuring irregular calcification and/or ossification at the center.

The risk of malignant transformation of osteochondromas remains below 1% [16]. Differential diagnosis with other neoplasms, such as chondrosarcoma, is necessary. It should be noted that chondrosarcoma can occur as a primary or secondary tumor to osteochondroma [17]. Certain specific signs should arouse suspicion of malignant transformation, including an increase in tumor size, the appearance of osteolysis, sharp exostosis contours, the presence of calcifications outside the predominantly ossified zone, erosion of the supporting or surrounding bone, thick cartilage cap (greater than 2 cm), and increased fixation on scintigraphy in adults. Detection of even one of these signs should prompt excision for carcinological management. Recurrence of exostosis is exceptionally rare and usually occurs when fragments of the cartilage cap remain after excision. For this reason, excision must be performed extraperiosteally. Moreover, recurrence should raise concerns about possible malignant transformation [17].

## Conclusions

Osteochondromas, prevalent benign bone tumors, usually manifest with cosmetic changes and symptoms arising from mechanical compression of adjacent structures. Any abrupt enlargement associated with pain should raise concerns about potential malignant transformation. Given the risk of sarcomatous degeneration, vigilant clinical and radiological surveillance is imperative, prompting systematic surgical intervention at the slightest suspicion.
